# Phylogenetic and Recombination Analysis of Clinical Vitreous Humor–Derived Adenovirus Isolates Reveals Discordance Between Serotype and Phylogeny

**DOI:** 10.1167/iovs.65.2.12

**Published:** 2024-02-06

**Authors:** Aaron W. Kolb, Viet Q. Chau, Darlene L. Miller, Nicolas A. Yannuzzi, Curtis R. Brandt

**Affiliations:** 1 Department of Ophthalmology and Visual Sciences, School of Medicine and Public Health, University of Wisconsin-Madison, Madison, Wisconsin, United States; 2 Bascom Palmer Eye Institute, University of Miami, Miami, Florida, United States; 3 McPherson Eye Research Institute, University of Wisconsin-Madison, Madison, Wisconsin, United States; 4 Department of Medical Microbiology and Immunology, School of Medicine and Public Health, University of Wisconsin-Madison, Madison, Wisconsin, United States

**Keywords:** human adenovirus, ocular, phylogenetics, virology, recombination, serology

## Abstract

**Purpose:**

To sequence, identify, and perform phylogenetic and recombination analysis on three clinical adenovirus samples taken from the vitreous humor at the Bascom Palmer Eye Institute.

**Methods:**

The PacBio Sequel II was used to sequence the genomes of the three clinical adenovirus isolates. To identify the isolates, a full genome-based multiple sequence alignment (MSA) of 722 mastadenoviruses was generated using multiple alignment using fast Fourier transform (MAFFT). MAFFT was also used to generate genome-based human adenovirus B (HAdV-B) MSAs, as well as HAdV-B fiber, hexon, and penton protein-based MSAs. To examine recombination within HAdV-B, RF-Net 2 and Bootscan software programs were used.

**Results:**

In the course of classifying three new atypical ocular adenovirus samples, taken from the vitreous humor, we found that all three isolates were HAdV-B species. The three Bascom Palmer HAdV-B genomes were then combined with over 300 HAdV-B genome sequences, including nine ocular HAdV-B genome sequences. Attempts to categorize the penton, hexon, and fiber serotypes using phylogeny of the three Bascom Palmer samples were inconclusive due to incongruence between serotype and phylogeny in the dataset. Recombination analysis using a subset of HAdV-B strains to generate a hybridization network detected recombination between nonhuman primate and human-derived strains, recombination between one HAdV-B strain and the HAdV-E outgroup, and limited recombination between the B1 and B2 clades.

**Conclusions:**

The discordance between serotype and phylogeny detected in this study suggests that the current classification system does not accurately describe the natural history and phylogenetic relationships among adenoviruses.

Human adenoviruses (HAdVs) are part the *Mastadenovirus* genus and are non-enveloped, linear, double-stranded DNA viruses with genomes of approximately 35 kilobases (kb). Since the 1950s,^1^^–^^6^ immunological (serology) and hemagglutinin studies of the three main structural proteins (penton, hexon, and fiber) have formed the basis of human adenovirus grouping. These serological-based analyses have progressed to polymerase chain reaction (PCR)-based serotyping techniques, followed by genome sequence–based phylogeny by the Human Adenovirus Working Group (http://hadvwg.gmu.edu/).^7^^–^^9^ Currently, HAdVs are grouped into seven species (A–G) with 114 genotyes designated by the Human Adenovirus Working Group using a haplotype-based system with a combination of the penton, hexon, and fiber serotypes.

Transmission of HAdVs regularly occurs by fecal–oral spread, aerosol inhalation, contaminated water, and direct conjunctival exposure.^10^^,^^11^ Human adenoviruses can cause respiratory, gastrointestinal, urinary, and ocular disease,^10^^,^^12^^–^^15^ as well as disseminated disease in immunosuppressed individuals.^16^^–^^21^ Adenoviral keratoconjunctivitis has been described since the late 1880s^22^^,^^23^; however, the viral causative agent was not isolated until 1943^24^ and was not identified as adenovirus until 1955.^25^ Ocular adenoviral disease usually presents in three forms; simple follicular conjunctivitis, pharyngoconjunctival fever (PFC), and epidemic keratoconjunctivitis (EKC).^26^ Three human adenovirus species (B, D, and E) are known to cause ocular infections^10^^,^^26^; serotypes B3, E4, and B7 are commonly associated with PFC and HAdV-D, and serotypes D8, D19, and D37 are linked with the more severe EKC.^26^^–^^28^ Although D8, D19, and D37 dominate EKC epidemiology, serotypes B3, E4, B7, D9, D53, and D54 have been recorded in surveillance studies.^29^^–^^32^ EKC is characterized by geographic epithelial keratitis, and multifocal subepithelial infiltrates can often develop which can affect visual acuity in chronic adenoviral keratitis.^33^^–^^35^ Although adenoviral ocular disease almost exclusively results in conjunctival and corneal pathology, a recent study detected human adenoviruses C and D in the vitreous humor of immunocompromised patients with uveitis and necrotizing retinitis.^36^

Adenovirus virions are icosahedral in structure with 20 facets, comprised of 12 hexon protein trimers and vertices made of five penton base proteins, with the vertices connected to the trimeric fiber attachment protein.^37^^–^^40^ In addition to these three main proteins are several minor proteins (e.g., IIIa, VI, VIII, IX) which appear to stabilize the capsid.^41^^–^^43^ Adenovirus primary attachment occurs when a cellular receptor binds the fiber protein knob. Human adenovirus fiber proteins bind a variety of cell surface molecules, with species A and C to G typically using the cellular coxsackievirus and adenovirus receptor,^44^^–^^49^ whereas HAdV-B fiber binds CD46, CD80, CD86, and desmoglein-2.^50^^–^^55^ Secondary attachment involves the penton base arginine–lycine–aspartate (RGD) motif typically binding host αvβ1, αvβ3, αvβ5, or α3β1 integrins.^56^^–^^59^

In the current study, we attempted to determine the genotype of three vitreous-origin adenovirus isolates using phylogenetics; however, the analysis was complicated by incongruences between serotypes and phylogenetics. Initial whole genome sequence analysis identified the three vitreous samples as HAdV-B. Fiber, hexon, and penton protein sequence–based phylogenetic investigation found that, in several cases, adenovirus serotypes do not form distinct phylogenetic units. A RF-Net 2–generated hybridization network comprised of 42 HAdV-B strains and a HAdV-E outgroup strain detected extensive recombination within B1 and B2 clades, as well as recombination between human and nonhuman primate–derived viral strains. Together, the data present a complex phylogenetic picture largely stemming from recombination and suggests that improvements in the current classification system are needed to more accurately reflect the evolutionary history and phylogenetic relationships of these viruses.

## Methods

### Vitreous Humor Adenoviral Isolates

Three intraocular adenovirus isolates were collected via vitrectomy in various clinical settings at the Bascom Palmer Eye Institute (BPEI) in Miami, Florida. The severity and nature of the ocular disease and immune status of source patients were not recorded. BPEI Clinical Microbiology Laboratory testing logs were evaluated to identify patients undergoing ocular tissue culture between 1993 and 2015. Approval from the University of Miami Institutional Review Board was obtained prior to conducting this study (protocol number: 20070960), which was performed in accordance with the Health Insurance Portability and Accountability Act of 1996 and adhered to the tenets of the Declaration of Helsinki. The National Center for Biotechnology Information (NCBI) accession numbers used in this study can be found in Supplementary Table S1. The raw sequencing *.bam files are available at NCBI Sequence Read Archive (PRJNA1055033). The multiple sequence alignments generated for this study are available for download at https://datadryad.org/stash/share/65SyCYLNBGaG10IqGhEpENpoA_fIuywYvSeTf3rPK64.

### Cells

To produce viral stocks and DNA for genomic sequencing, A549 cells (CCL-185; American Type Culture Collection, Manassas, VA, USA) were used. The cells were grown in Dulbecco's Modified Eagle's Medium (DMEM), with 10% fetal bovine serum and antibiotics.

### Viral DNA Purification

To isolate adenovirus DNA, 10 confluent TC100 plates of A549 cells were infected with viral stock and DMEM + 2% serum. The infected cells were monitored daily for cytopathic effect (CPE) and harvested 24 hours after the monolayer reached 100% CPE. The harvested cells underwent centrifugation at 600*g* for 10 minutes. The resulting pellet was combined with 5 mL of supernatant and then underwent three freeze–thaw cycles. The freeze–thawed lysate and supernatant were combined and then centrifuged at 600*g* for 10 minutes. The resulting supernatant was pipetted onto a 36% sucrose cushion (in PBS), followed by centrifugation for 80 minutes at 24,000*g*. Following centrifugation, the supernatant was aspirated, and the remaining pellet was resuspended in 3 mL of TE buffer (10-mM Tris, pH 7.4; 1-mM EDTA) in addition to 3-M sodium acetate (0.15-M final concentration, pH 5.5). RNase A (50 µg/µL) was added to the preparation, which was then incubated for 30 minutes at 37°C. Proteinase K (50 µg/µL) and SDS (0.1%) were added to the viral preparation, followed by a 30-minute incubation at 37°C. Phenol and chloroform extractions were then performed to purify the DNA. Viral DNA was precipitated by adding two volumes of ice-cold 95% ethanol, followed by desalting with 70% ethanol. Finally, the purified adenoviral DNA was resuspended in sterile water.

### Genome Sequencing

The genomic sequencing of the adenovirus isolates was performed as previously described.^60^ Briefly, the extracted DNA was quantified using a Qubit dsDNA High Sensitivity Kit (Thermo Fisher Scientific, Waltham, MA, USA). The size and quality of the DNA were assessed using the Femto Pulse electrophoresis system (Agilent, Santa Clara, CA, USA). A Pacific Biosciences Microbial Multiplex library (PN 101-696-100 v07 instructions) was then generated, followed by DNA shearing with g-TUBES (Covaris, Woburn, MA, USA). The Femto Pulse system was utilized to determine the library quality and was quantified with the Qubit dsDNA High Sensitivity Kit. The PacBio Sequel II (PacBio, Menlo Park, CA, USA) was used to sequence the viral DNA library with one single-molecule real-time sequencing cell with the Sequel Polymerase Binding Kit 2.2 at the University of Wisconsin-Madison Biotechnology DNA Sequencing Facility. The subsequent raw PacBio reads were filtered and processed by CCS calling (CSS 6.2.2; https://github.com/PacificBiosciences/ccs), then demultiplexed. Hifiasm^61^ was used to assemble the demultiplexed reads into contigs and then Mega7^62^ to manually assemble the contigs into genomes.

### Multiple Sequence Alignments

Prior to phylogenetic and recombination analysis, the genomes of 722 adenovirus strains were downloaded from GenBank (Supplementary Table S1). The genomes of the 722 isolates were combined with the three Bascom Palmer adenovirus isolates, and a genome-based multiple sequence alignment (MSA) was generated using multiple alignment using fast Fourier transform (MAFFT), version 7.45.^63^ Next, a HAdV-B whole genome MSA comprised of 327 sequences was created. Additional fiber, penton, and hexon protein sequence–based MSAs of 315 adenovirus B isolates (plus the three Bascom Palmer samples) were produced. An MSA comprised of adenovirus B fiber nucleotide sequences was also generated using MAFFT. MAFFT was also used to produce a full genome-based MSA comprised of a subset of 42 total adenovirus B isolates for RF-Net 2–based recombination analysis (see below). For the adenovirus B MSAs, adenovirus E isolate 4482 (accession MT771648) was used as an outgroup.

### Phylogenetic and Clade Cutoff Analysis

To conduct phylogenetic analysis, the optimal nucleotide substitution and related parameters for a given multiple sequence alignment were determined using IQ-TREE 2.^64^ Phylogenetic networks were generated using SplitsTree 4.15.1,^65^ in conjunction with parameters defined by IQ-TREE 2. Species-level and intraspecies clade cutoffs were established using a delimiting method that has been previously used with alphaherpesviruses.^66^^,^^67^ Depending on the dataset, either whole genome or individual protein multiple sequence alignments were used for clade delimiting. Briefly, for the nucleotide-based datasets, maximum composite likelihood pairwise distances were calculated using Mega7,^62^ and amino acid–based pairwise distances were calculated using the Jones–Taylor–Thornton (JTT)^68^ substitution model. The pairwise distances were then graphed as histograms and overlayed with kernel density plots using the R 4.2.1-arm64^69^ (R Foundation for Statistical Computing, Vienna, Austria) and RStudio 2022.07.2-576^70^ software packages. Species and clade cutoffs (if possible) were determined by finding the trough between the low and high pairwise distance populations.

### Recombination Analysis

Recombination analysis was examined using two methods. First, RDP4^71^ was used by imputing a genome-based multiple sequence alignment and a sliding window of 1800 base pairs (bp), 500-bp steps, 500 bootstrap replicates, and the Jin and Nei substitution model.^72^ A hybridization network was inferred for adenovirus B viruses by first generating an MSA consisting of a subsampling of 42 HAdV-B and one HAdV-E (outgroup) genomes. A subsampled dataset was used due to computational limitations. Using the full MSA dataset would result in excessively high computation time and would be beyond the ability of the software to optimize parameters. The dataset subsample included isolates from the main adenovirus B clades, with adenovirus E as an outgroup. The MSA of the subsampled dataset was then broken into 21 1800-bp partitions, and maximum-likelihood trees (1000 bootstrap replicates) were generated for each partition using IQ-TREE 2. The phylogenetic trees generated by IQ-TREE 2 (*.treefile) were then entered into RF-Net 2^73^ to produce hybridization networks. The number of reticulations (recombination events) for RF-Net 2 was optimized by producing embedding cost graphs at 5, 10, 15, 20, 25, 30, and 40 reticulations and determining at which point the embedding cost curve began to flatten. When the optimal number of reticulations had been determined, RF-Net 2 was run a total of 10 times using the “-e” and “-f” options (embed and fast, respectively). Embedding cost dynamic graphs were generated for each run, and hybridization networks were visualized using IcyTree.^74^ Reticulations recorded in 70% of the RF-Net 2 runs were determined to be valid. Mega7 was used to visualize the phylogenetic topology dataset by producing a consensus tree from the partitioned maximum-likelihood trees produced by IQ-TREE 2. The valid reticulations were then plotted onto the consensus tree.

## Results

### Genome Sequencing and Assembly

Three adenovirus strains were isolated from the vitreous humor of patients suspected to be infected with adenovirus at the BPEI. The three isolates were propagated in A549 cells, the DNA was isolated, and the genomes were sequenced using the PacBio Sequel II. The sequenced genomes were assembled using hifiasm, resulting in genome lengths varying from 34,652 bp for BP-AdV2 to 35,199 bp for BP-AdV1 (Table 1). Average coverage for the genomes ranged from 210× for BP-AdV2 to 25,776× for BP-AdV3 (Table 1).

**Table 1.** Bascom Palmer Adenovirus Sequencing Results

Virus Isolate	Number of Reads	Average Read Length	Average Coverage	Genome Length
BP-AdV1	54,251	8670	6288×	35,199 bp
BP-AdV2	10,608	8517	210×	34,652 bp
BP-AdV3	346,794	10,004	25,776×	34,735 bp

### Whole Genome Phylogenetic Analysis of the Adenovirus Isolates

**Figure 1.**
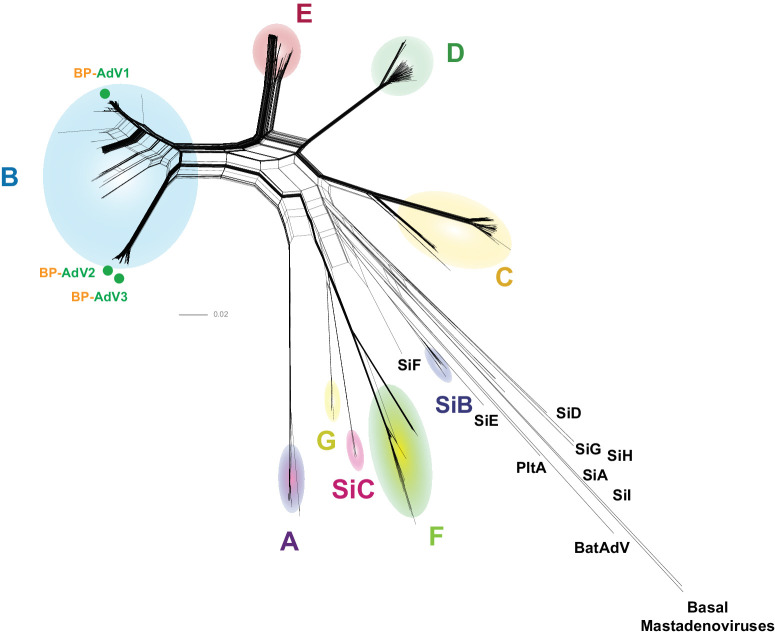
The phylogenetic position of the three vitreous humor BPEI adenovirus isolates among *Mastadenovirus*. The genome sequences of the three Bascom Palmer adenoviruses were combined with the genomes of 722 mastadenoviruses in an MSA. A phylogenetic network was generated from the MSA using SplitsTree,^65^ with optimized settings (GTR+G+I; *P_inv_* = 0.026; gamma = 0.864) determined by IQTREE-2.^64^ The phylogenetic positions of the three Bascom Palmer isolates is denoted by *green dots*.

**Figure 2.**
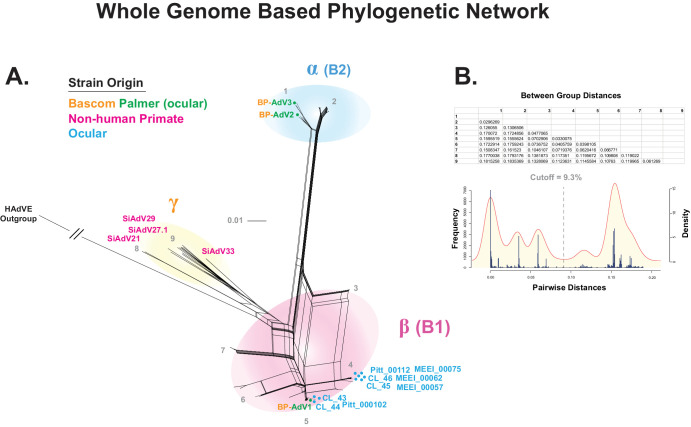
Whole genome-based phylogenetic network of HAdV-B and distance-based clade cutoff. The genome sequences of the three Bascom Palmer isolates were combined with the genome 315 HAdV-B strains (plus HAdV-E outgroup) to generate an MSA using MAFFT. (**A**) SplitsTree was used to produce a phylogenetic network using the MSA, with optimized parameters (GTR+G+I; *P_inv_* = 0.363; gamma = 0.623) determined by IQTREE-2. (**B**) Pairwise distance-based histogram and superimposed kernel density plot (KDP) that were generated in R. The trough between the KDP peaks resulted in a clade distance cutoff of 9.3%. Nine potential clades were tested and the between-group mean distances are reported in the table. Three group distances were above 9.3% and determined to be valid clades, labeled α, β, and γ.

**Figure 3.**
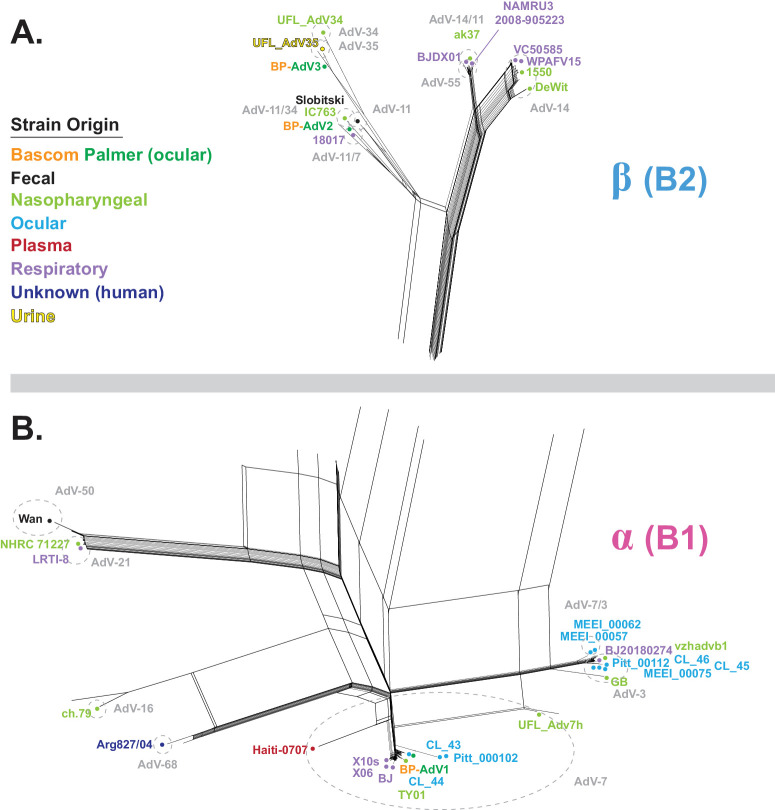
Enlarged views of the α (B1) and β (B2) clades from the whole genome–based HAdV-B phylogenetic network. (**A**) Enlargement of the B2 clade. (**B**) Enlargement of the B1 clade from Figure 2. A subset of HAdV-B strains is colored according to origin: Bascom Palmer (*orange* and *green*), fecal (*black*), nasopharyngeal (*light green*), ocular (*light blue*), plasma (*red*), respiratory (*lilac*), unknown (*dark blue*), and urine (*yellow*). Genotype groups are labeled in *light gray* with groups enclosed by *dotted*, *light-gray circles*.

To identify the species of the three Bascom Palmer vitreous adenovirus isolates, an MSA was first produced, comprised of the genomes of the three vitreous isolates plus 722 *Mastadenovirus* genome sequences (Supplementary Table S1). A *Mastadenovirus* phylogenetic network was generated that placed all three Bascom Palmer isolates within HAdV-B (Fig. 1). To further define the phylogenetic position of the Bascom Palmer isolates within HAdV-B, a whole genome– based MSA was created that included 323 HAdV-B genomes and HAdV-E as an outgroup. A new MSA was generated to ensure alignment accuracy among the HAdV-B strains, and removing non-HAdV-B sequences was performed to aid in downstream analysis. HAdV-E was chosen as the outgroup because it was the adenovirus species closest to the HAdV-B in Figure 1. From this MSA, a phylogenetic network was generated that placed BP-AdV1 within the B1 group and BP-AdV2 and BP-AdV3 within the B2 group (Fig. 2). We next wanted to determine if human adenovirus B strains phylogenetically group together based on anatomical origin of isolation, given that ocular HAdV-B strains are not well studied. To accomplish this, fecal, nasopharyngeal, plasma, respiratory, urine, and all currently sequenced ocular-derived^75^ isolates were identified in an expansion of the B1 and B2 clusters (Figs. 3A, 3B). Figures 3A and 3B show that the respiratory and nasopharyngeal isolates were generally evenly distributed across the B1 and B2 clusters. Contrasting this, the nine non–Bascom Palmer ocular isolates formed two groupings in the B1 cluster, corresponding to AdV-3 and AdV-7 serotypes. Although the adenovirus B serotypes generally formed coherent phylogenetic groupings, an inconsistency between serotype and phylogeny was observed in the ak37 strain, which is designated as an AdV-11/14 serotype mix; however, it grouped with AdV-55 serotype strains (Fig. 3A).

Rather than assigning HAdV-B clades subjectively (for example, exclusively visually), we decided to use an objective method to establish clades. To accomplish this, we used a pairwise distance–based clade delimiting method^66^^,^^67^^,^^76^^–^^78^ to test nine potential clades based on the topology of the Figure 2 split network. A distance cutoff of 9.3% was determined as the lowest trough between the two major kernel density plot peaks (Fig. 2B). The between-group distances of the potential nine clades were compared to see if the values were above or below the 9.3% distance cutoff. For example, potential clades 3 and 4 were 4.77% distant, placing these groups within the same clade; however, the distance between groups 2 and 3 was 13.06%, resulting in placing these groups in separate clades. Using this method, three clades were recovered, which we termed α, β, and γ, with α corresponding to the common nomenclature B1, β to B2, and γ to nonhuman primate B strains (Fig. 2).

### Fiber Protein Analysis

**Figure 4.**
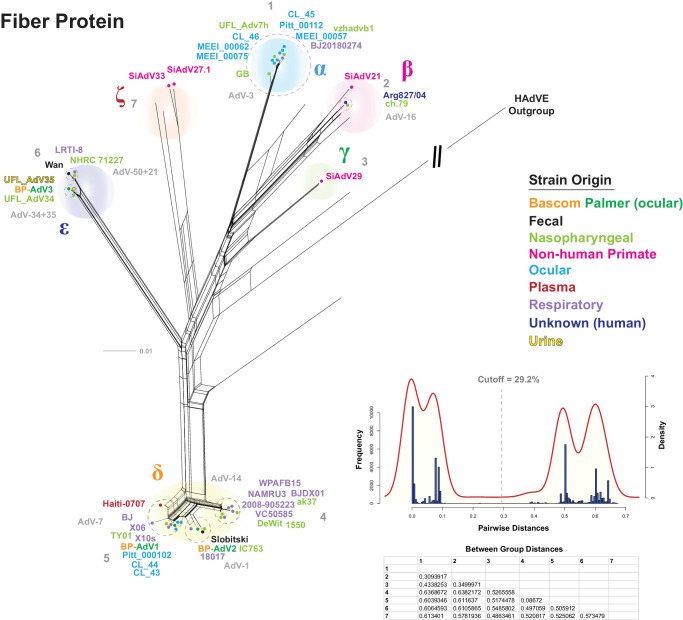
HAdV-B fiber protein phylogenetic network and distance-based clade cutoff. A multiple sequence alignment comprised of three Bascom Palmer and 320 HAdV-B fiber protein sequences (plus HAdV-E outgroup) was generated and used to produce a phylogenetic network with SplitsTree. The optimized network parameters (JTT+G+I; *P_inv_* = 0.114; gamma = 1.464) were calculated using IQTREE-2. A subset of HAdV-B strains is colored according to origin: Bascom Palmer (*orange* and *green*), fecal (*black*), nasopharyngeal (*light green*), nonhuman primate (*pink*), ocular (*light blue*), plasma (*red*), respiratory (*lilac*), unknown (*dark blue*), and urine (*yellow*). Serotype groups are labeled in *light gray* with groups enclosed by *dotted*, *light-gray circles*. A pairwise distance-based histogram and KDP were generated in R, and a clade distance cutoff of 23.2% was determined as the midpoint between the KDP peaks. Seven possible clade groups were tested with the between-group distances (calculated in Mega 7^62^) shown in a table below the KDP. This analysis recovered six clades: α, β, γ, δ, ε, and ζ.

Serotyping adenovirus fiber, hexon, and penton proteins has been the standard method of categorizing different strains; however, this results in a limited phylogenetic picture. Because fiber, hexon, and penton serotyping is so widely used, we first wanted to determine both the fiber amino acid sequence–based phylogenetic position of the three Bascom Palmer isolates and conduct a full phylogenetic analysis of HAdV-B fiber. To do this, an MSA was produced using 326 HAdV-B fiber amino acid sequences which was then used to generate a phylogenetic network (Fig. 4). A fiber nucleotide sequence–based phylogenetic network was constructed and yielded nearly identical results (Supplementary Fig. S1). Next, a distance-based clade delimiting method, as described above, was used to test seven possible clades based on the topology of the phylogenetic network and resulted in a distance cutoff of 29.2%. After comparing the between-group distance values, six clades were recovered: α, β, γ, δ, ε, and ζ (Fig. 4). Bascom Palmer BP-AdV1 and BP-AdV2 isolates grouped into the δ clade with the AdV-7 and AdV-11 serotypes, respectively, whereas BP-AdV3 grouped into the ε clade. Further examination of the phylogenetic network revealed that all of the ocular isolates aside from BP-AdV3 clustered into the α and δ clades, with nearly all corresponding to the AdV-3 and AdV-7 serotypes (Fig. 4). The majority of the respiratory isolates clustered into the δ clade, whereas the nasopharyngeal isolates were generally evenly distributed among the α, β, δ, and ε clades. Clade ζ was comprised exclusively of nonhuman primate HAdV-B strains (Fig. 4).

The fiber protein ε clade contains two sets of phylogenetically indistinguishable serotypes. One ε clade branch includes the nearly phylogenetically identical AdV-50 serotype Wan strain and two AdV-21 serotype strain sequences (Fig. 3). The other ε clade branch is comprised of the phylogenetically indistinguishable AdV-34 and AdV-35 serotype sequences (Fig. 3). Additionally, incongruences between whole genome and fiber protein phylogeny were observed. Human adenovirus serotype 7 strains were grouped into B1 using whole genome data; however, fiber protein sequence analysis showed a close phylogenetic relationship between this group, AdV-11 (B2) and AdV-14 (B2), which comprised the fiber δ clade (Figs. 3, 4). The fiber β clade included the nonhuman and human SiAdV-21 and HAdV-16 (B1) virus strains.

### Hexon Protein Analysis

**Figure 5.**
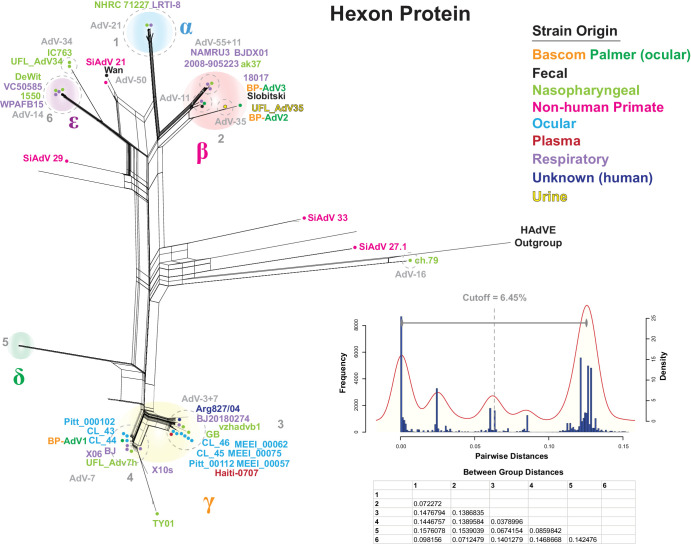
HAdV-B hexon protein phylogenetic network and distance-based clade cutoff. A multiple sequence alignment comprised of three Bascom Palmer and 320 HAdV-B hexon protein sequences (plus HAdV-E outgroup) was generated and used to produce a phylogenetic network with SplitsTree. The optimized network parameters (JTT+G+I; *P_inv_* = 0.625; gamma = 0.748) were calculated using IQTREE-2. A subset of HAdV-B strains is colored according to origin: Bascom Palmer (*orange* and *green*), fecal (*black*), nasopharyngeal (*light green*), nonhuman primate (*pink*), ocular (*light blue*), plasma (*red*), respiratory (*lilac*), unknown (*dark blue*), and urine (*yellow*). Serotype groups are labeled in *light gray* with groups enclosed by *dotted*, *light-gray circles*. A pairwise distance-based histogram and KDP were generated in R, and a clade distance cutoff of 6.45% was determined as the midpoint between the KDP peaks. Six possible clade groups were tested with the between-group distances (calculated in Mega 7) shown in a table below the KDP. This analysis recovered five clades: α, β, γ, δ, and ε.

Similar to the fiber protein analysis, a phylogenetic examination of the hexon protein was performed, which included 324 HAdV-B strains. A clade delimiting procedure, similar to what was performed above, recovered five clades (α, β, γ, δ, and ε), with a distance cutoff of 6.45% (Fig. 5). A hexon protein sequence–based phylogentic network (Fig. 5) showed reticulations forming two “poles,” such that the “north pole” contained the α, β, and ε clades, and the “south pole” included the γ and δ clades. Two of the vitreous origin Bascom Palmer isolates (BP-AdV2 and BP-AdV3) grouped into the β clade, and one (BP-AdV1) sorted into the γ clade (Fig. 5). Apart from the two β-clade Bascom Palmer strains, all of the ocular isolates grouped into the γ clade, correlating with the AdV-3 and AdV-7 serotypes. The nasopharyngeal and respiratory strains contrast this, as they were evenly distributed among the different hexon clades. Interestingly, the AdV-50 Wan and SiAdV-21 strains displayed an intermediate position between the α (AdV-21) and ε (AdV-14) clades. The ch.79 (AdV-16) hexon sequence additionally showed a close phylogenetic relation to that of the HAdV-E outgroup (Fig. 5). In the hexon β clade, serotype 11 strains appeared to form two groups. A core AdV-11 group included reference strain Slobitski, and a second group contained hexon serotypes 11 and 55 (Fig. 5). Unlike the fiber protein phylogeny, the serotype 34 and 35 hexon sequences occupied different clades: ε and β, respectively. A possible phylogeny and serotype incongruency was observed in the γ clade, where the AdV-7 genotype Haiti-0707 hexon sequence grouped with the AdV-3 serotyped strains, such as strain GB.

### Penton Protein Analysis

**Figure 6.**
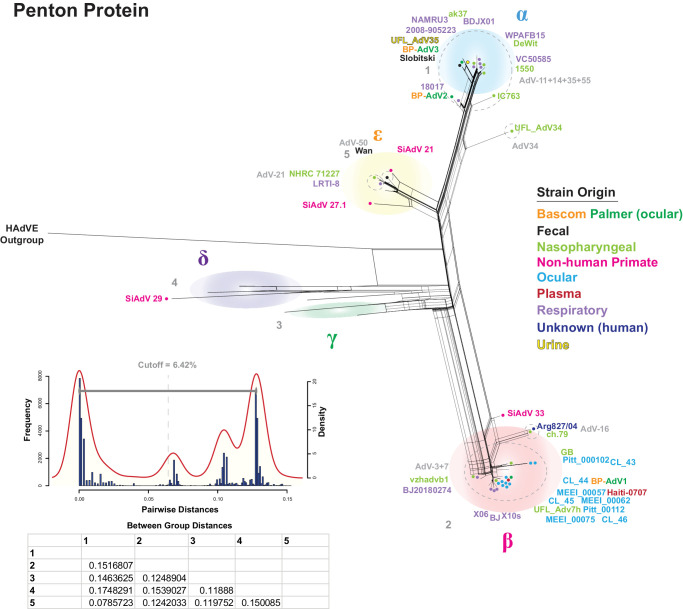
HAdV-B penton protein phylogenetic network and distance-based clade cutoff. A multiple sequence alignment comprised of three Bascom Palmer and 320 HAdV-B penton protein sequences (plus HAdV-E outgroup) was generated and used to produce a phylogenetic network with SplitsTree. The optimized network parameters (JTT+G+I; *P_inv_* = 0.325; gamma = 0.306) were calculated using IQTREE-2. A subset of HAdV-B strains is colored according to origin: Bascom Palmer (*orange* and *green*), fecal (*black*), nasopharyngeal (*light green*), nonhuman primate (*pink*), ocular (*light blue*), plasma (*red*), respiratory (*lilac*), unknown (*dark blue*), and urine (*yellow*). Serotype groups are labeled in *light gray* with groups enclosed by *dotted*, *light-gray circles*. A pairwise distance-based histogram and KDP were generated in R, and a clade distance cutoff of 6.42% was determined as the midpoint between the KDP peaks. Five possible clade groups were tested with between-group distances (calculated in Mega 7) shown in a table below the KDP. This analysis recovered five clades: α, β, γ, δ, and ε.

Next, an MSA of 323 penton protein adenovirus sequences was produced, and pairwise distance clade delimiting was performed. Five possible clades were tested, and a cutoff distance of 6.42% resulted in the recovery of all possible five clades (α, β, γ, δ, and ε) (Fig. 6). A phylogenetic network based on the penton MSA showed that BP-AdV2 and BP-AdV3 grouped into the α clade, which was comprised of AdV-11, AdV-55, and AdV-35 serotypes (Fig. 6). The remaining Bascom Palmer ocular isolate, BP-AdV1, was placed into the β clade, which included the remaining ocular isolates. Nonhuman primate HAdV-B strains formed the γ and δ clades, and SiAdV-21 and SiAdV-27.1 strains were included in the otherwise human adenovirus ε clade (Fig. 6). Multiple incongruencies between phylogenetic position and serotype were found; for example, in penton clade β, HAdV-3 and HAdV-7 viruses grouped closely together and were not reliably distinguished phylogenetically. A similar result can be seen in the α clade, where AdV-11, AdV-14, AdV-35, and AdV-55 did not form reliably phylogenetically distinct units (Fig. 6).

### Recombination Analysis

Although phylogenetic networks such as those produced by SplitsTree^65^ are useful in assessing recombination in a dataset, they do not explicitly detect the genomic segments being recombined or the directionality. The commonly used Bootscan^79^^,^^80^ algorithm is highly effective in identifying recombined segments; however, drawbacks exist, with one being that a single sequence at a time can be scanned as the reference, and data interpretability can be compromised with larger datasets. Explicit networks that identify horizontal gene transfer and hybridization events or recombination events (i.e., reticulation events) in conjunction with phylogenetic trees are commonly referred to as hybridization networks. To identify possible recombination in the three vitreous origin Bascom Palmer adenoviruses as well as HAdV-B strains in general, a hybridization network was generated using the RF-Net 2 algorithm.^73^ A representative dataset consisting of 42 HAdV-B genomes (plus HAdV-E outgroup) (Table 2) was used due to computational limitations (specifically, computation time), as well as data interpretability.

**Table 2.** Human Adenovirus B Subset Strain Accession Number, Genotype, and Serotype Summary

Strain	Accession Number	Genotype	Penton	Hexon	Fiber	Phylogenetic/Serotype Incongruence (+/−)
BP-AdV1	OR669096	—	—	—	—	−
BP-AdV2	OR669098	—	—	—	—	−
BP-AdV3	OR669097	—	—	—	—	−
1550	MG905106.1	AdV-14	14	14	14	+
18017	KT970440.1	—	11	11	7	+
ak37	JX423385.1	AdV-11a	14	11	14	+
Arg827/04	JN860678.1	AdV-68	16	3	16	−
BJ	MH355567.1	AdV-7	7	7	7	+
BJ20180274	MW748649.1	—	—	—	—	+
BJDX01	MK886831.1	AdV-55	—	—	—	+
ch.79	AY601636.1	AdV-16	—	—	—	−
CL43	KF268134.1	AdV-7	7	7	7	+
CL44	KF268125.1	AdV-7	7	7	7	+
CL45	KF268132.1	AdV-7	3	7	3	+
CL46	KF268128.1	AdV-3	3	3	3	+
deWit	MG905111.1	AdV-14	14	14	14	+
GB	NC_011203.1	AdV-3	3	3	3	+
Haiti-0707	MN531562.1	AdV-7	—	—	—	+
IC763	KF906413.1	AdV-34+11	—	34	11	+
LRTI-8	KY307858.1	AdV-21a	—	—	—	+
MEEI_00057	KF268210.1	AdV-3	7	3	3	+
MEEI_00062	KF268212.1	AdV-3	7	3	3	+
MEEI_00075	KF268202.1	AdV-3	3	3	3	+
NAMRU3_2008-905223	MN654384.1	AdV-55	—	—	—	+
NHRC_71227	KJ364584.1	AdV-21	—	—	—	+
Pitt_00102	KF429748.1	AdV-7	7	7	7	+
Pitt_00112	KF429752.1	—	3	3	7	+
SiAdV_21	AC_000010.1	—	—	—	—	−
SiAdV_27.1	FJ025909.1	—	—	—	—	−
SiAdV_29	FJ025916.1	—	—	—	—	−
SiAdV_33	FJ025908.1	—	—	—	—	−
Slobitski	NC_011202.1	AdV-11	11	11	11	−
TY01	KP896480.1	AdV-7	—	—	—	+
UFL-AdV7h	KF268126.1	AdV-7	7	7	3	+
UFL-AdV34	KF268196.1	AdV-34	34	34	34	+
UFL-AdV35	KF268124.1	AdV-35	35	35	35	+
VC50585	KY021428.1	AdV-14	14	14	14	+
vzhadvb1	MH828478.1	—	—	—	—	+
Wan	AY737798.1	AdV-50	—	—	—	+
WPAF15	MG642745.1	AdV-14	—	—	—	+
X06	MG696133.1	AdV-7	—	—	—	+
X10s	MG736304.1	AdV-7	—	—	—	+

**Figure 7.**
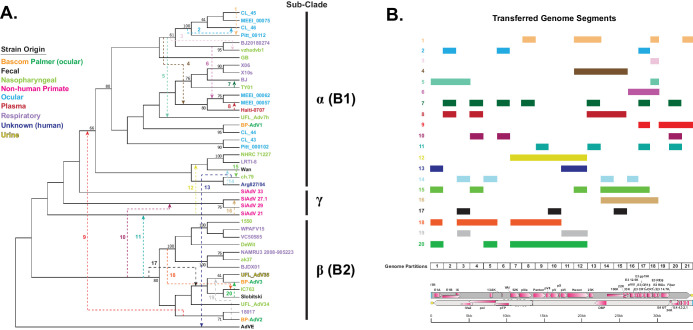
Hybridization network and corresponding transferred genome segments of a subset of 42 HAdV-B strains. (**A**) The hybridization network. (**B**) The transferred segments including a genome map. In the hybridization network, HAdV-B strains are colored according to origin: Bascom Palmer (*orange* and *green*), fecal (*black*), nasopharyngeal (*light green*), nonhuman primate (*pink*), ocular (*light blue*), plasma (*red*), respiratory (*lilac*), unknown (*dark blue*), and urine (*yellow*). To generate the hybridization network, a genomic MSA of a subset of 42 HAdV-B (plus HAdV-E outgroup) was divided into 21 1800-bp partitions (shown near the bottom of **B**). For each partition, ML trees (1000 bootstraps) were generated with IQTREE-2. The data obtained were entered into RF-Net 2, which was set for 40 possible reticulations (recombination events) and run 10 times. Reticulations occurring in 70% of the runs were deemed valid. This analysis returned 20 reticulations, which are numbered with multiple colors on a ML consensus tree. *Arrows* show the directionality of the genetic transfer. Panel **B** shows the matching, color-coded transferred genome segments of the 20 reticulations from panel **A**. Partition and corresponding adenovirus genome maps are shown at the bottom of panel **B**.

Briefly, the analysis was performed by partitioning or splitting the 43-strain genomic multiple sequence alignment into 21 1800-bp partitions or genomic segments. RF-Net 2 differs from Bootscan in that Bootscan utilizes a sliding window, but RF-Net 2 uses partitioned genome segments. Optimized maximum-likelihood (ML) trees were generated for each partition using IQ-TREE 2.^64^ The partition size of 1800 bp was chosen because this size reflected a balance among resolution, computational capabilities, and data interpretability. The IQ-TREE 2 ML tree output was entered into RF-Net 2 to generate hybridization networks. The maximum detected number of possible recombination (reticulation) events is set by the user in RF-Net 2; however, this can be optimized by plotting the embedding cost per number of reticulations. The embedding cost is the measurable error derived from improperly generated tree networks; thus, when the embedding cost curve flattens, the optimal number of reticulations has likely been established. The number of RF-Net 2 input reticulations was optimized by graphing the embedding cost dynamic of RF-Net2 runs with 5, 10, 15, 20, 25, 30, and 40 reticulations to determine where the embedding cost curve begins to flatten. This analysis resulted in curve flattening at approximately 40 reticulations (Supplementary Fig. S2). In our use, the hybridization networks generated by RF-Net 2 varied slightly per run, so to address this RF-Net 2 was run 10 times and set to detect 40 possible reticulations. This resulted in 10 hybridization networks (Supplementary Fig. S3) and 10 embedding cost dynamic graphs (Supplementary Fig. S4). Detected reticulations for each RF-Net 2 run were recorded (Supplementary Table S2) and ranged from 32 reticulations for run 3 to 37 reticulations for run 6. The variability of RF-Net 2 run results may be indicative of optimization issues within the RF-Net 2 program. To deal with RF-Net 2 output variability, reticulations detected in 70% of the runs were deemed as valid (Supplementary Table S2). This analysis yielded a total of 20 reticulations, which were then mapped to a phylogenetic consensus tree for visualization purposes (Fig. 7A). The recombined genomic partition blocks for each reticulation are shown in Figure 7B.

The hybridization network showed extensive recombination within the B1 and B2 clades; however, only one reticulation (number 9) was recorded between B1 and B2 strains (Fig. 7A). Recombination between HAdV-B serotypes was detected by the RF-Net 2 analysis; for example, reticulations 4, 5, and 6 show recombination between serotypes 3 and 7, and reticulation 15 denotes recombination between serotypes 50 and 16. Three reticulations (Fig. 7A, numbers 10, 11, and 12) were detected between human adenovirus B strains and nonhuman primate (chimpanzee derived; see Supplementary Table S1) B strains. Interestingly, one adenovirus interspecies reticulation event (reticulation number 13) was identified between the HAdV-E outgroup and the HAdV-B ch.79 strain, corresponding to E1A (partition 1) and hexon (partitions 11 and 12). Three recursive reticulations, which are reticulations between a root and a strain deriving from that root, were also detected (numbers 2, 3, and 17) and may be indicative of rate variation.

**Figure 8.**
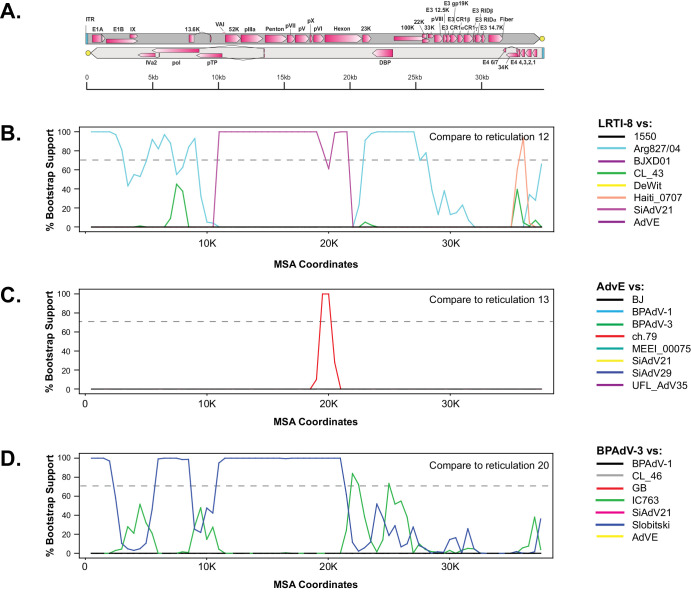
Proof-of-principle representative bootscans of three reticulations detected by RF-Net 2. (**A**) An adenovirus genome map is depicted at the top of the figure. (**B**–**D**) Panel **B** corresponds to RF-Net 2 reticulation 12, **C** to reticulation 13, and **D** to reticulation 20. The strains used in each bootscan analysis are color coded and found to the right of each panel. The *gray dotted line* in each bootscan represents 70% bootstrap support. Each bootscan was performed using RDP4 with a window size of 1800 bp, step size of 500 bp, 500 bootstrap replicates, and the Jin and Nei nucleotide substitution model.^72^

Because the RF-Net 2 method is relatively new and to our knowledge has not yet been applied to adenovirus datasets, we also performed a small number of recombination bootscans as proof of principle. The first bootscan performed scanned the HAdV-E outgroup against eight HAdV-B strains, including ch.79 (Fig. 8B). This bootscan validated reticulation number 9 (Fig. 7) by detecting a signal corresponding to hexon from ch.79 (Fig. 8B), which also corresponds to the hexon protein phylogenetic analysis in Figure 5. Additional bootscans (Figs. 8B, 8C) scanning BP-AdV3 and LTRI-8 strains, respectively, validated recombined partition blocks in reticulations 15 and 10 (Fig. 7B) by recovering similar genomic areas in the bootscans.

## Discussion

Next-generation sequencing has revolutionized virology as with many branches of biological science, resulting in hundreds of viral genomes being deposited into GenBank, including adenoviruses. Genomic DNA sequences are now being used to determine adenovirus genotyping and serotyping (http://hadvwg.gmu.edu/). In the current study, we analyzed the genomes of three vitreous humor–derived adenovirus isolates in the context of other HAdV-B genomes in an attempt to determine the serotype/genotype. In the process of this analysis, we found inconsistencies between human adenovirus B serotyping and phylogenetics.

### Origin of the Viral Isolates

The three adenovirus isolates described in this study were collected in a clinical setting at the BPEI from the vitreous humor of patients experiencing severe posterior pathology, with suspicion of a viral infection. The fact that the Bascom Palmer–derived adenoviral samples were collected via vitrectomy is highly unusual but not unprecedented, as recent reports have identified human adenovirus–induced uveitis and retinitis.^36^^,^^81^ Unfortunately, it does not appear that detailed records were taken of either ocular disease manifestations or the immune status of the Bascom Palmer source patients. Regardless, between the current study and two recent case studies, it is unclear how human adenovirus was present in the posterior eye segment. Human adenoviruses have a strong tropism for the corneal surface and are nearly exclusively known for causing pathology to the cornea and surrounding conjunctiva.^26^^,^^82^^–^^84^ Contrasting this, several human adenovirus–based vectors, including those utilizing the HAdV-35 (species B) fiber protein, have been used in gene therapy–related studies to transduce different retinal cell types,^85^^–^^87^ suggesting the capacity of some adenovirus strains to infect the retina. These findings suggest that adenoviruses may cause acute retinal necrosis. Further studies are needed to determine if adenovirus-related uveitis and retinitis reports are either exceptional events or clinically underdiagnosed.

### Identification and Genome Phylogenetics of the Viral Isolates

Following sequencing, the genomes of three Bascom Palmer isolates were combined in a MSA with over 700 adenovirus genomes and identified as human adenovirus B (Fig. 1). This result was somewhat surprising because, although species B has been known to cause ocular infections, it has not been associated with severe keratoconjunctivitis, which is caused by human adenovirus species D.^26^^,^^33^^,^^82^^,^^88^ Between the current study and Sugita et al.,^36^ five total vitreous-related adenovirus samples have been identified as human adenovirus species B, C, or D.

Following the identification of the three Bascom Palmer isolates as HAdV-B, the genome sequences from these isolates were combined with more than 300 HAdV-B genomes in a phylogenetic network to refine phylogenetic placement (Fig. 2). Two of the Bascom Palmer samples grouped into subclade B1, and the remaining sample grouped into subclade B2. Efforts to assign a genotype to the samples were not successful due to ambiguities and inconsistencies detected between phylogeny and serotype, which are discussed below.

Further analysis, which included the nine ocular genomic sequences available from GenBank, showed that the ocular HAdV-B isolates (except for BP-AdV2 and BP-AdV3) formed two groups in subclade B1. This phylogenetic grouping pattern of the HAdV-B ocular isolates extended into fiber, hexon, and penton protein–based phylogenetic networks (Figs. 4^5^–6). Due to the relatively small sample size of ocular HAdV-B samples, the significance of these results is unclear, especially compared to nasopharyngeal- and respiratory-derived strains, which were generally evenly distributed across the B1 and B2 subclades (Fig. 3). Should future phylogenetic analysis show close grouping of ocular HAdV-B isolates, it may suggest a common genetic component underlying ocular strains.

### Phylogeny and Serotype

Since the 1950s, human adenovirus strains have been conventionally classified by penton, hexon, and fiber protein serology and hemagglutination-based techniques.^1^^–^^6^^,^^89^ Current human adenovirus genotyping and serotyping have shifted to genomic sequence–centered criteria recommended by the Human Adenovirus Working Group. This is predicated on the concept that serological groups are comprised of distinct phylogenetic units. Our analysis, which included over 300 genomic HAdV-B sequences and to our knowledge is the most comprehensive to date, found that, although broadly the different serological groups do form phylogenetic units, this is not always true. For interpretability of the data figures, a subset of HAdV-B strains was chosen, with the accession numbers, genotypes, and serotypes shown in Table 2. An example of phylogenetic ambiguity was found between the AdV-34 and AdV-35 serotype fiber sequences (Fig. 4). The AdV-34 and AdV-35 fiber protein sequences differ by only two amino acids, at positions 207 and 217, and are essentially phylogenetically indistinguishable. It is curious that the AdV-34 and AdV-35 fiber proteins are able to be distinguished by serotyping as the predicted fiber structures are also highly similar (Supplementary Fig. S5). Additionally, the AdV-3 and AdV-7 penton serotypes failed to recover a consistent phylogenetic structure (Fig. 6). Figure 6 shows that the reference AdV-3 strain GB (β clade) was phylogenetically distinct from the AdV-7 strains; however, the remaining AdV-3 penton serotype strains were not distinguishable from the AdV-7 strains. Similarly, in penton α clade, penton serotype 11 strain 18017 is phylogenetically distinct from serotype 11 strain 1550, which groups with serotype 14 strains.

The most likely explanation for the serotype and phylogenetic ambiguity or discordance is that the antibody epitopes are restricted to a small number of amino acids and do not reflect the full phylogenetic signal of the protein sequences or viral genomes, which itself can be complicated by recombination. Another factor affecting these results is likely the nature of the large dataset incorporating over 300 strains in the analysis. Most earlier serotyping and phylogenetic examinations of human adenoviruses have used a relatively low number of strains or focused on a single serotype.^90^^–^^93^ A lower number of sequences in an analysis can give simplistic and ultimately incorrect results when compared to a large dataset, as has been observed with human herpesvirus type 1 (HSV-1).^60^^,^^94^ As expected from previous studies with adenovirus strains,^83^^,^^93^^,^^95^^–^^98^ we found evidence of recombination in the three Bascom Palmer isolates.

**Figure 9.**
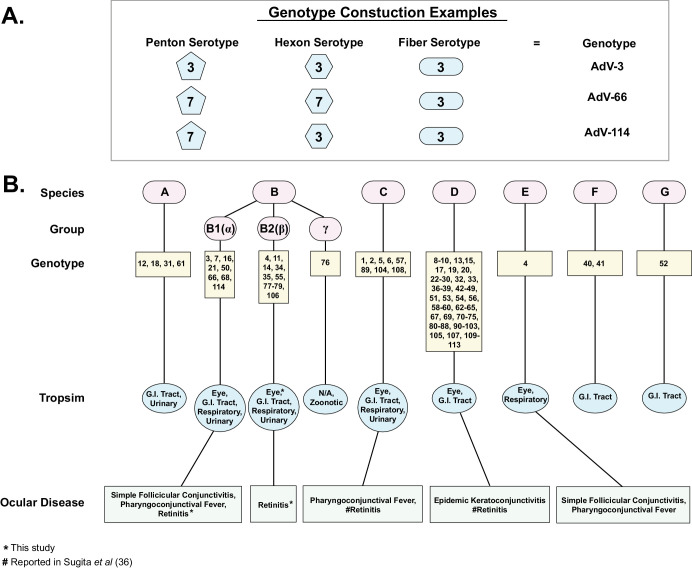
Example of human adenovirus genotype determination by serotype, plus a summary of human adenovirus species and genotypes with associated tropism and disease. (**A**) Illustration of how different combinations of penton, hexon, and fiber serotypes are used to determine new genotypes. (**B**) Adenovirus species A to G are shown with derivative genotypes, tropisms, and ocular disease phenotypes. The current study reports the first instances of adenovirus B1 and B2 with retinitis. *Retinitis associated with HAdV-B first reported in the current study; #retinitis associated with HAdV-C and HAdV-D previously reported in Sugita et al.^36^

An initial question about the observation of discordance between phylogeny and serotype is what impact, if any, is there in a clinical setting. Human adenovirus genotypes are determined by a combination of penton, hexon, and fiber serotypes, with different serotype combinations potentially resulting in new genotype designations, as depicted in Figure 9A. Figure 9B summarizes the human adenovirus genotypes, tropism, and ocular disease phenotypes and shows (for example, in HAdV-B) that the genotype designations correspond to the B1 (α), B2 (β), and γ subgroups, and B1 viruses are more likely to cause ocular disease. Although some genotypes are more likely to be associated with an ocular disease phenotype (e.g., AdV-3 and AdV-7 with pharyngoconjunctival fever),^99^^,^^100^ they are not necessarily predictive of clinical outcomes. Because serotyping only captures differences in variable epitopes in three proteins,^7^^,^^101^ plus our observation that serotype and phylogeny may not harmonize beyond identifying the human adenovirus species, serotyping may provide limited information for clinicians. Moreover, continued genotyping is potentially confusing; for example, the new AdV-114 is simply a recombinant of AdV-3 and AdV-7 (Fig. 9A). A genome sequence–based classification, such as the PANGO classification system (https://cov-lineages.org/index.html) recently devised for SARS-CoV-2,^102^ may be more informative both for epidemiology and the clinic.

### Recombination Analysis

Phylogenetic trees are extensively used to explore evolutionary relationships using either nucleotide or protein sequences. Errors in inferring relationships can occur when different parts of a given sequence have different evolutionary histories (for example, due to recombination), which can result in non-treelike behavior. One way of dealing with non-treelike behavior is to use “implicit” techniques, such as split networks, as generated by SplitsTree. The SplitsTree program detects disparate phylogenetic signals in a dataset; however, the internal nodes of split networks do not represent ancestral taxa nor are possible recombined sequences identified and thus are “implicit.”^65^^,^^103^ Most viral recombination analyses use the effective Bootscan^79^ algorithm of the RDP4 software program^98^^,^^104^^–^^106^ to identify recombined sequences and breakpoints; however, it can be negatively affected by more than a handful of samples, and it does not address evolutionary relationships. A second way to deal with non-treelike behavior is to use phylogenetic hybridization networks, which capture recombination (reticulation events), and the network nodes represent actual ancestral taxa.^65^^,^^107^ One recent, relatively easy to use hybridization network method is RF-Net 2,^73^ which allows a bird's-eye view of recombination within the dataset. Extensive recombination in adenoviruses has been well established; however, here we introduce a previously unused tool to the adenovirus field (RF-Net 2), which identified recombination events (reticulation events), the genomic segments that were transferred, the directionally of the transfer, and a phylogenetic tree. Due to software dataset optimization and computation time limitations, we used a representative dataset of 42 HAdV-B strains instead of the full 300+. The resulting hybridization network detected 20 reticulations (recombination events), of which only one was between the B1 and B2 subclades, with most of the detected recombination occurring within the subclades (Fig. 7). Intriguingly, three human- and nonhuman primate–derived HAdV-B strain reticulations were detected, plus one reticulation event between the HAdV-E outgroup and an AdV-16 genotype strain (Fig. 7). This result suggests that zoonotic adenovirus infections may be more prevalent than previously thought. Although the RF-Net 2 analysis was performed with a relatively small dataset, these human adenovirus species B recombination patterns would not have been observed using the bootscan program alone. Further, the hybridization network analysis underscores the contribution recombination plays in the phylogenetic complexity of human adenovirus strains and its role as a mechanism that can lead to incongruence between phylogeny and serotype.

### Application to Other Human Adenovirus Species

The focus of the current study is HAdV-B, and one primary question is whether the current findings of discordance versus phylogeny apply to the remaining human adenovirus species. The current results from HAdV-B suggest that recombination is the primary cause of the discordance between serology and phylogeny. Recombination has been shown to be pervasive in human adenoviruses C and D^98^^,^^108^^,^^109^; however, recombination between serotypes within species A and F may be more restricted,^110^^,^^111^ and species E contains only one serotype. Although detected recombination between serotypes may be restricted in species A and F, adenovirus replication and recombination appear to be linked,^97^ implying that recombination occurs, even if undetected. Based on the link between replication and recombination, for each human adenovirus species potential discordance between serotype and phylogeny exists and is likely to be identified as an increasing number of strains are isolated and their genomes sequenced.

## Conclusions

In conclusion, our initial goal of identifying the genotype for each of the three vitreous humor adenovirus strains using phylogenetics was obfuscated by incongruence between serotype and phylogeny in the dataset. The initial analysis identified the three clinical adenovirus isolates as HAdV-B, and subsequent genome sequence–based analysis with over 300 HAdV-B genomic sequences showed phylogenetic clustering of HAdV-B ocular isolates into the B1 subclade. Dissonance between phylogeny and serotype was detected in the dataset; for example, penton serotypes 3 and 7 were phylogenetically indistinguishable. Recombination analysis detected extensive recombination within the B1 and B2 subclades and recombination between human and nonhuman primate strains. The use of serology to classify adenovirus isolates does not necessarily reflect the true genomic relationships or evolutionary history of this family of viruses, resulting in some confusion. The detected discrepancies are likely to be caused by the relatively small set of amino acids sampled by serological methods, recombination between strains, and that serology only samples three viral genes. Together these findings suggest that an improvement to the adenovirus strain classification system may be useful to the adenovirus field.

## Supplementary Material

Supplement 1

Supplement 2

Supplement 3
