# Supplementary material for: Phylogenetic and Recombination Analysis of Clinical Vitreous Humor–Derived Adenovirus Isolates Reveals Discordance Between Serotype and Phylogeny
Source: Invest Ophthalmol Vis Sci. 2024 Feb 6;65(2):12. doi: 10.1167/iovs.65.2.12 (PMC10854415; doi:10.1167/iovs.65.2.12)
Supplement: Supplement 1 [file iovs-65-2-12_s001.pdf]

Supplementary Figure 1.

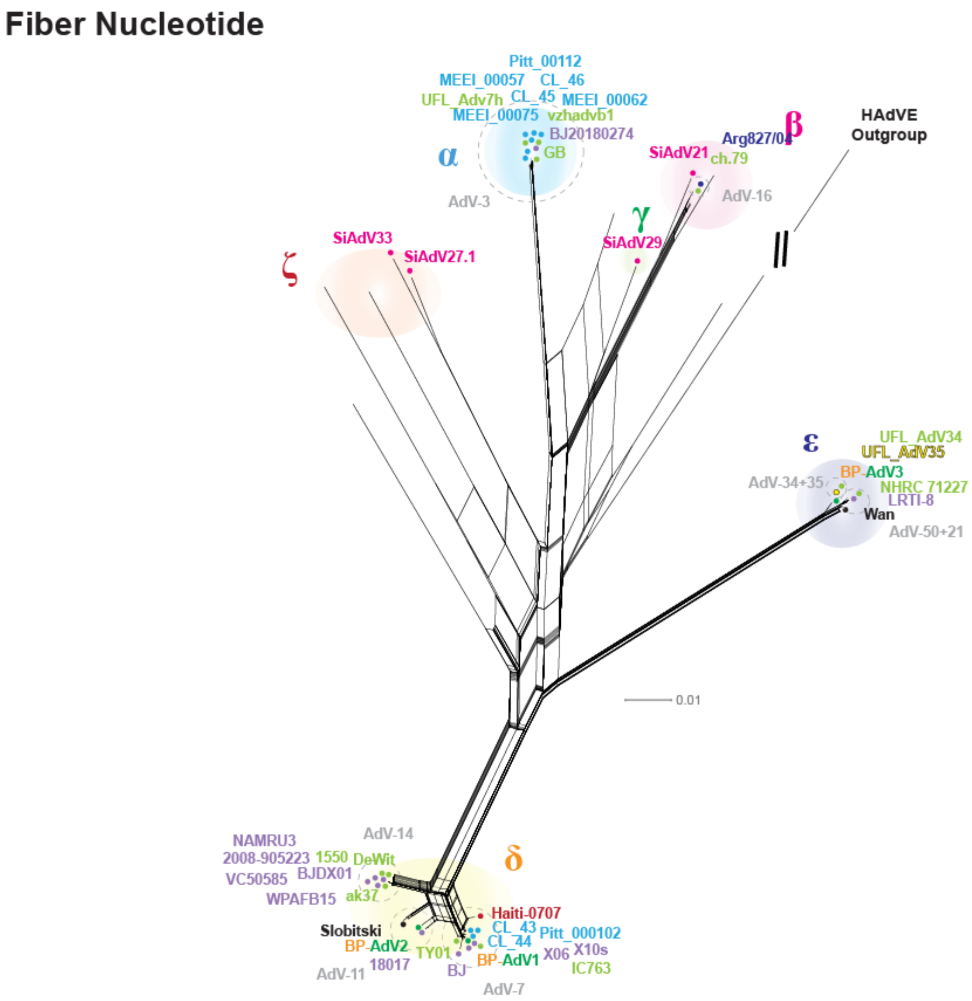

Supplementary Figure 1. HAdV-B fiber nucleotide-based phylogenetic network. A multiple sequence alignment comprising 3 Bascom Palmer and 320 HAdV-B fiber nucleotide sequences (plus HAdV-E outgroup) was generated and used to produce a phylogenetic network with Splitstree. The optimized network parameters (GTR+G+I; p-inv = 0.140, gamma = 1.423) were calculated using IQTREE-2. A subset of HAdV-B strains were colored according to origin; Bascom Palmer (orange and green), fecal (black), nasopharyngeal (light green), non-human primate (pink), ocular (light blue), plasma (red), respiratory (lilac), unknown (dark blue) and urine (yellow). Serotype groups are labeled in light gray with groups enclosed by dotted, light gray circles. Subclades α, β, γ, δ, ε, and ζ were carried over from the fiber protein analysis in Figure 4.

Supplementary Figure 2.

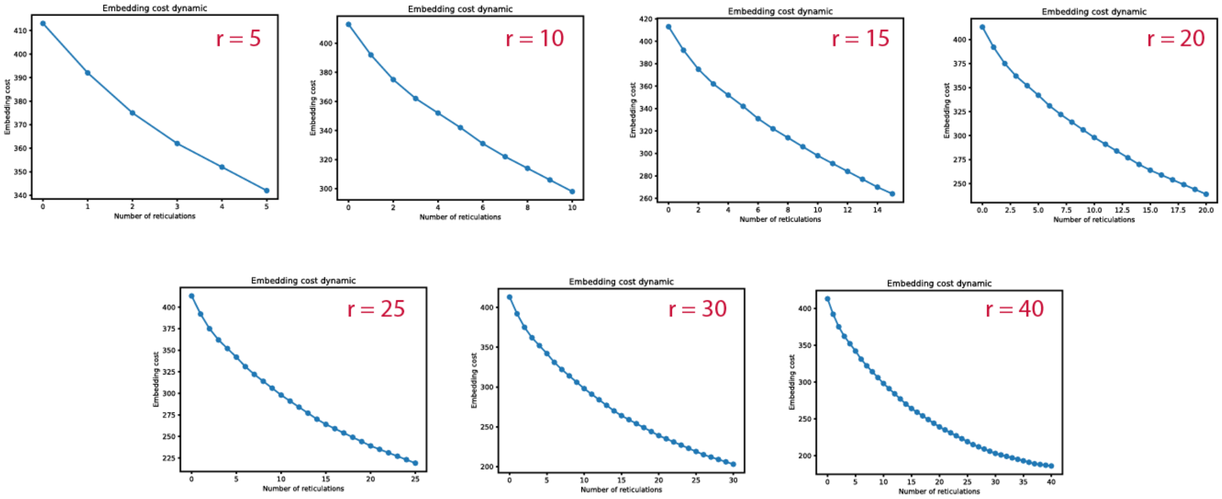

Supplementary Figure 2. Embedding cost graph dynamic for RF-NET 2 set to 5, 10, 15, 20, 25, 30 and 40 reticulations. The embedding cost dynamic curve begins to flatten at 40 reticulations.

Supplementary Figure 3.

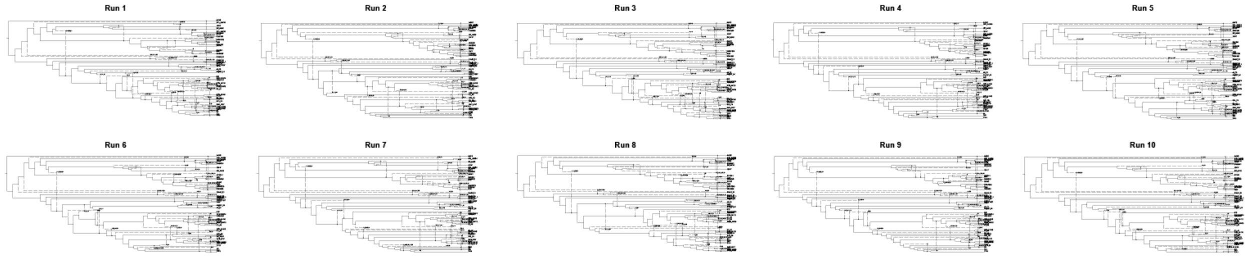

Supplementary Figure 3. The maximum likelihood trees with reticulations (dotted lines with arrows) and transferred genome partitions for each of the 10 RF-NET 2 runs.

Supplementary Figure 4.

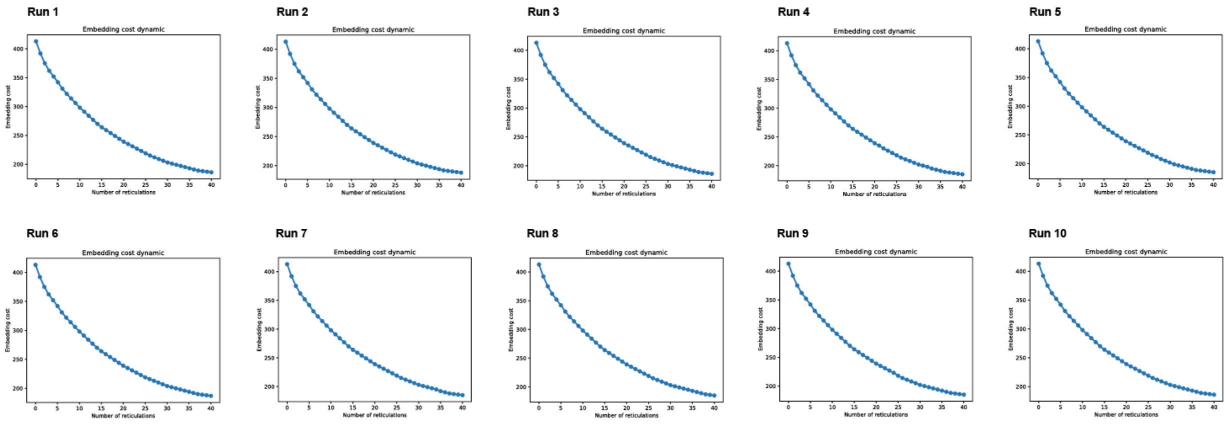

Supplementary Figure 4. Embedding cost dynamic graphs for each of the 10 RF-NET 2 runs (set to 40 reticulations).

Supplementary Figure 5.

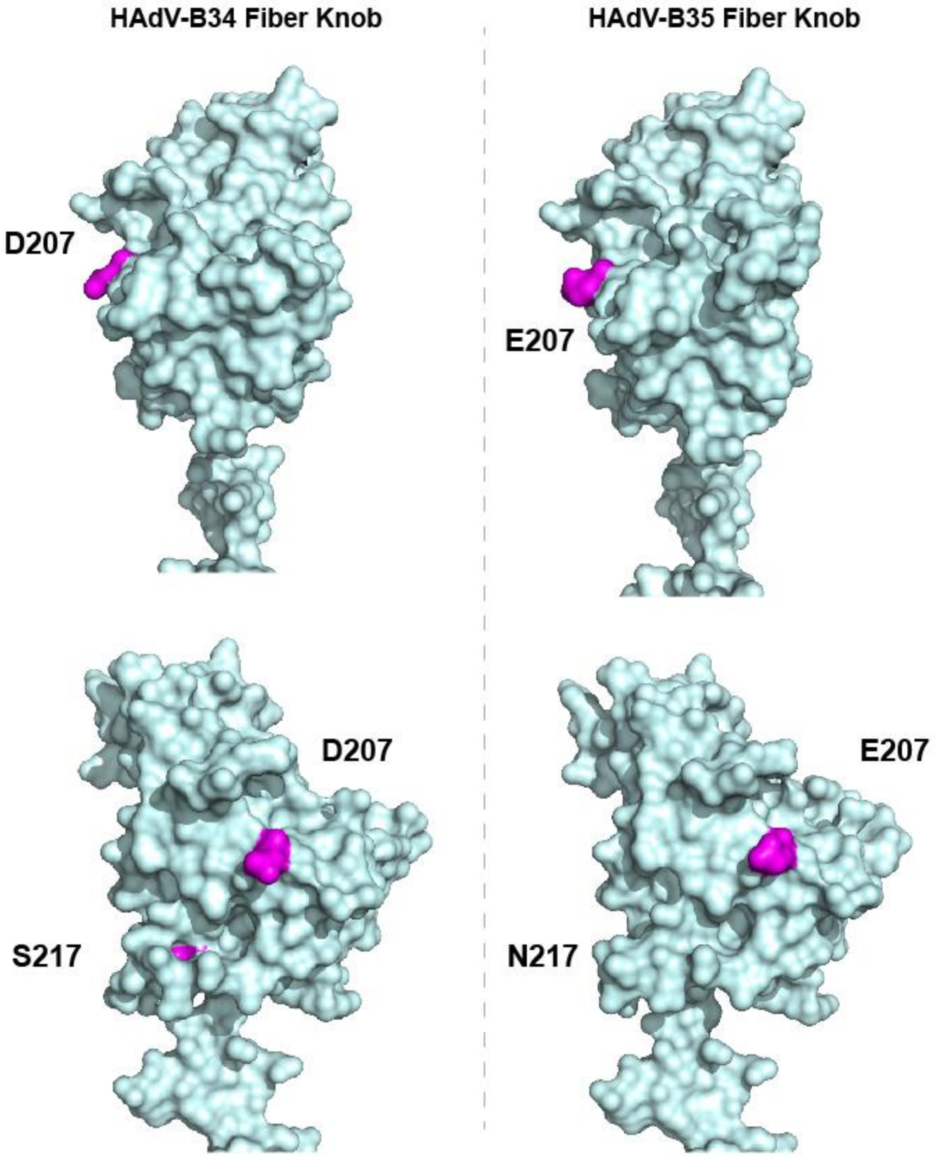

Supplementary Figure5. Predicted protein structures of the HAdV-B34 and B35 fiber knobs using Alphafold2. Fiber protein knob amino acid differences between HAdV-B34 and B35 are highlighted in magenta with corresponding single letter amino acid and site number.
